# Is the Pathogenic Ergot Fungus a Conditional Defensive Mutualist for Its Host Grass?

**DOI:** 10.1371/journal.pone.0069249

**Published:** 2013-07-10

**Authors:** Pauliina P. Wäli, Piippa R. Wäli, Kari Saikkonen, Juha Tuomi

**Affiliations:** 1 Department of Biology, University of Oulu, Oulu, Finland; 2 Kolari Unit, Finnish Forest Research Institute, Kolari, Finland; 3 Plant Production Research, MTT Agrifood Research Finland, Jokioinen, Finland; Indian Institute of Science, India

## Abstract

It is well recognized, that outcomes of mutualistic plant-microorganism interactions are often context dependent and can range from mutualistic to antagonistic depending on conditions. Instead, seemingly pathogenic associations are generally considered only harmful to plants. The ergot fungus (*Claviceps purpurea*) is a common seed pathogen of grasses and cereals. Ergot sclerotia contain alkaloids which can cause severe toxicity in mammals when ingested, and thus the fungal infection might provide protection for the host plant against mammalian herbivores. Theoretically, the net effect of ergot infection would positively affect host seed set if the cost is not too high and the defensive effect is strong enough. According to our empirical data, this situation is plausible. First, we found no statistically significant seed loss in wild red fescue (*Festuca rubra*) inflorescences due to ergot infection, but the seed succession decreased along increasing number of sclerotia. Second, in a food choice experiment, sheep showed avoidance against forage containing ergot. Third, the frequency of ergot-infected inflorescences was higher in sheep pastures than surrounding ungrazed areas, indicating a protective effect against mammalian grazing. We conclude that, although ergot can primarily be categorized as a plant pathogen, ergot infection may sometimes represent indirect beneficial effects for the host plant. Ergot may thus serve as a conditional defensive mutualist for its host grass, and the pathogenic interaction may range from antagonistic to mutualistic depending on the situation.

## Introduction

Interactions between plants and microorganisms have traditionally been characterized based on the often visible primary effect of the microbe on plant fitness. Pathogens clearly deplete host resources and act as harmful antagonists for the plant. Mutualists, such as certain endophytes and mycorrhizas, offer the host some beneficial service outweighing the consumption of the host resources. These relationships are not straightforward in nature, and the conditional characteristics of symbiotic (in the sense of living together) species associations are well recognized today. The presumed mutualists may not always be advantageous for host fitness, and the total fitness effects may vary from beneficial to antagonistic depending on the conditions [1]–[5]. By contrast, the possible conditional aspect of seemingly pathogenic interactions is much less discussed.

Mutualistic effects of mainly antagonistic associations have previously been suggested in relation to plant adaptations to herbivory, for grazing may improve plant fitness through overcompensation in some situations (for discussion and references, see [6]–[8]). Some animal-parasite interactions have also been shown to turn beneficial for the host in certain special conditions (rev. in [9]). Similar situations are likely in plant-pathogen interactions as well, and several possible factors could contribute to such an alternative outcome. Firstly, a pathogen may have additional and often diverse subsidiary effects on its host (direct or indirect, acting on host physiology or ecology [10]) that can be difficult to uncover [4]. On the other hand, the manifestation and impact of the effects on host fitness may vary depending on conditions (i.e. interacting genotypes, other interacting species and the growth conditions of the system) [1], [3], [5], [11], [12]. Moreover, a host's compensation and even overcompensation for primary cost may alter the initial situation [13].

The ergot fungus, *Claviceps purpurea* (Fr.) Tul., is a common seed pathogen of temperate grasses and cereals. Ergot infects single grass florets and develops a fungal tissue called a sclerotium instead of a grass seed. Ergot is defined as a plant pathogen because it depletes the host's resources and causes direct seed loss to the host plant (e.g., [14], [15]).

However, ergot sclerotia contain a variety of alkaloids, many of which are toxic to mammals. Ingestion of ergot within grass or grain products can cause severe and eventually lethal intoxication in both cattle and humans. Accordingly, ergot has been responsible for serious poisoning epidemics in human history and is still a cause of economic losses in grain production, especially because the sclerotia have to be removed from the infected seed sets (rev. in [16]–[18]). Several plant inhabiting fungi, like grass endophytes, have been suggested to play a part in the herbivore defence of their host plants [19], [20]. In such a case, the decrease in the palatability of a plant to herbivores is often due to toxic metabolites produced by the fungus, representing a form of 'acquired chemical defence' [21]. Grass grazing mammals have previously been shown to avoid eating grasses infected with endophytic fungi that produce ergot alkaloids (e.g., [22]). Although ergot is usually considered merely as a pathogen, such a protective effect has been speculated to occur also with ergot [17], [19], [23]–[25].

In this study, we present (1) a simple model showing how the costs of ergot infection, in terms of lost viable seeds, may be balanced by protective effects against grazing. Second (2), we present empirical data for the support of the model. The costs of ergot infection are estimated with seed data on wild red fescue (*Festuca rubra* L. sl.). The demonstration of the actual protective effect of ergot infection is done via sheep grazing: We compared the abundance of ergot on red fescue inside and outside sheep pastures. If the infection has protective effects on the host plant, ergot infected inflorescences should be less frequently eaten and therefore relatively more abundant inside the pastures. Further, we present experimental results on domestic sheep food choice in a situation where animals were provided ergot-free and, alternatively, ergot-containing forage.

### Predictions: how does ergot influence plant fitness?

We consider a condition where ergot infection would be favourable to its host by comparing the average fitnesses of ergot-infected and non-infected plants. Because ergot is toxic to mammalian grazers, it is highly likely that ergot influences the risk of grazing on the host plant. It is well-known that the presence of a toxic plant can decrease grazing risk of its neighbours [26]–[28]. Accordingly, we can propose a hypothesis that a seed infected by ergot may provide associational defence for other seeds in the ergot-infected inflorescence. If so, then the net effects of ergot on host fitness, relative to a non-infected plant, depends on how many seeds it will lose due to the infection (cost of infection) and how many it will save due to reduced seed predation.

**Figure 1:**
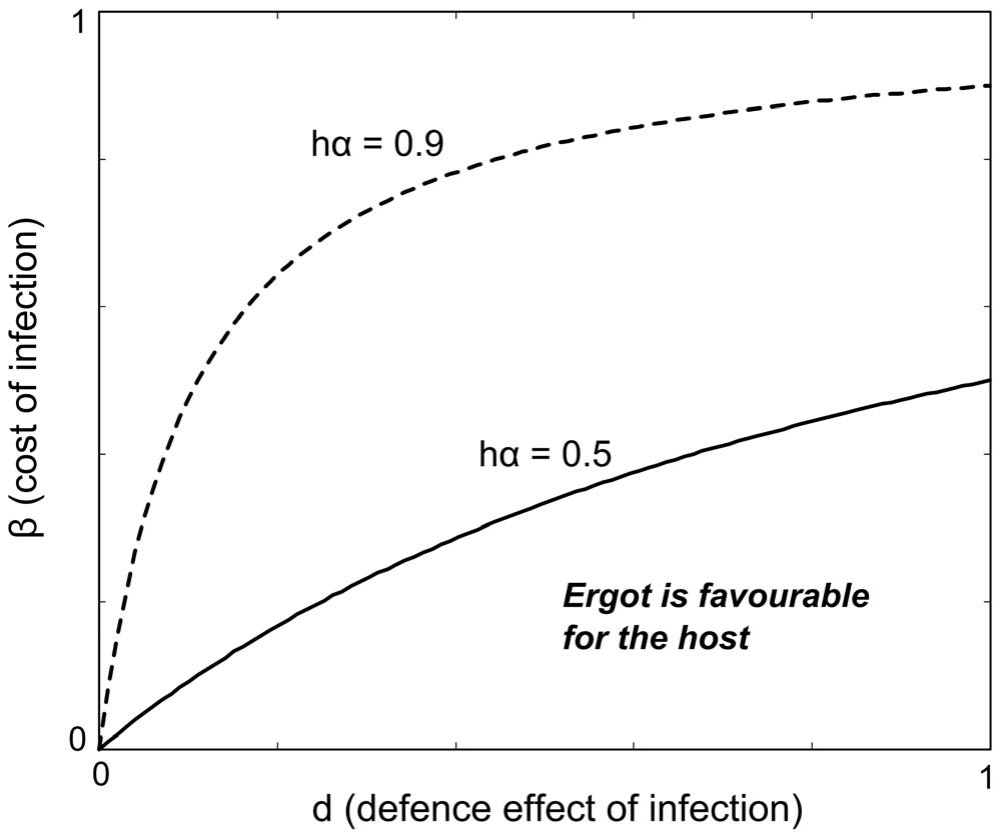
Effect of ergot on host fitness. Parameter space where ergot infection has a positive (below the lines) or negative (above the lines) net effect on host fitness. β = relative loss of seeds due to ergot infection, d = protective effect of ergot infection, hα = cost of herbivory.

Assume that B is the average number of seeds per plant in the absence of grazing and fungal infection. If h is the risk of grazing (0≤h≤1) and α is the relative decrease in seed number per plant when being grazed (0≤α≤1), the expected fitnesses of non-infected and infected plants will be B(1-hα) and B(1- β)[1-hα(1-d)], respectively, where β denotes the relative decline in viable seeds per plant if infected by ergot (0≤β≤1), and d is the relative decline in the risk of grazing (h) if the plant has been infected by ergot (0≤d≤1). The infection will be beneficial for the host if infected plants have on average higher fitness than non-infected, or otherwise expressed as,
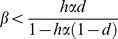



Thus, ergot can benefit the plant if the cost of infection is low enough and ergot infection has sufficiently high defensive effect against seed predation (Figure 1).

**Figure 2:**
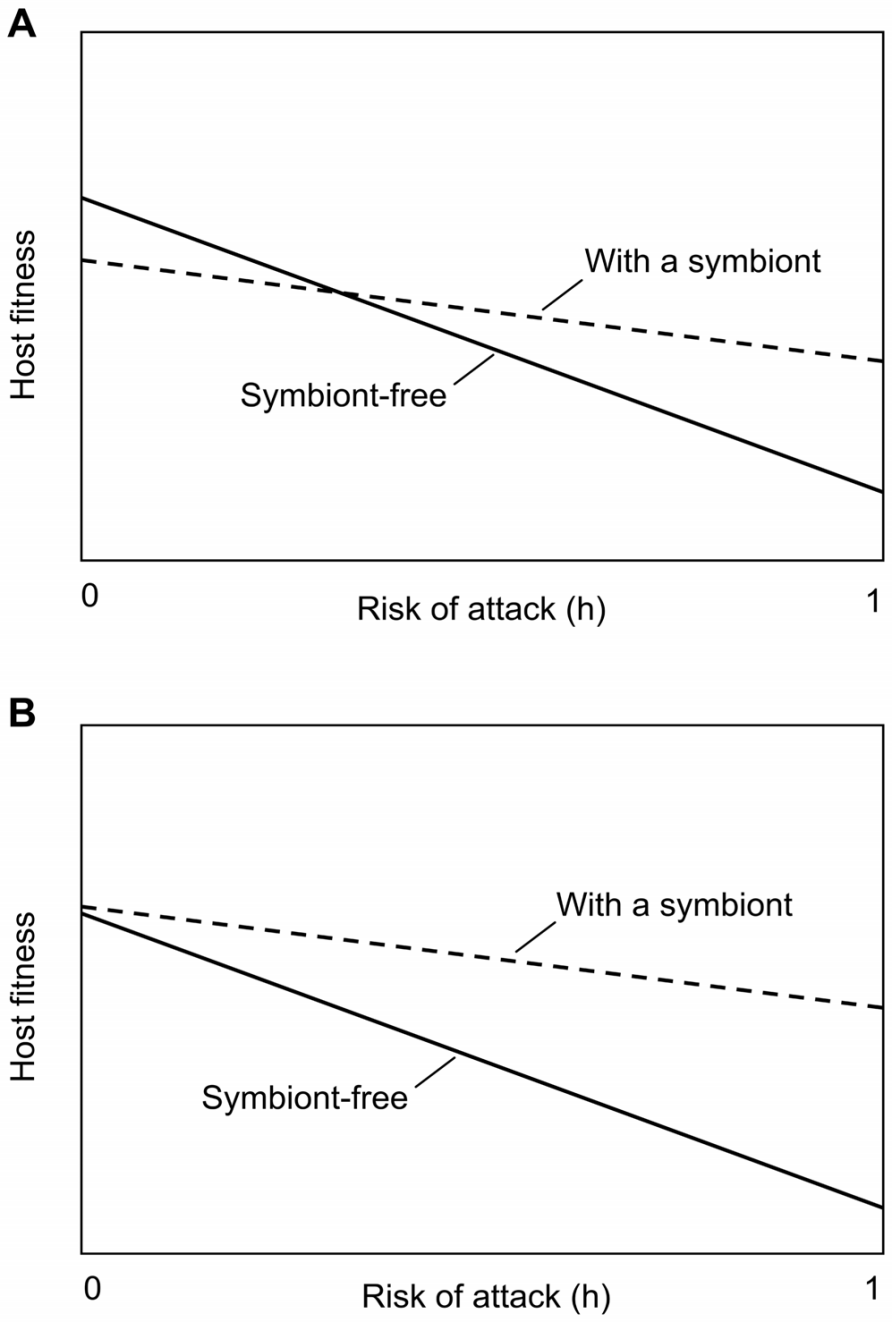
Host fitness and context dependency in defensive symbiosis. Average host fitness with or without a protective symbiont in relation to risk of attack. Defensive mutualism assumes that the symbiont improves host fitness in the presence of the enemy (h = 1), however the symbiont itself may (A) or may not (B) incur a fitness cost for the host in the absence of the enemy (h = 0).

This theoretical argument implies two main questions concerning the fitness consequences of ergot on the host plant. First, is low cost of infection a feasible assumption in the wild? Secondly, is there any evidence that ergot could have a defence effect in favour of the host plant? If the answers are positive, it is possible that the ergot-plant relationship could represent a case of “defensive mutualism”, generally defined as a mutually beneficial relationship between two species in which one protects the other from an enemy that causes a fitness loss to the latter (e.g., [19]). In defensive mutualism, the species that provides protection may or may not itself incur a net fitness cost in response to the other (Figure 2A and 2B, accordingly). In the case of grass-fungi interactions, the first alternative (Figure 2A) would correspond to a costly pathogen and the other one (Figure 2B), for example, to an asexual *Epichloë* endophyte that causes no visible or apparent costs to its host. In the first scenario the symbiont itself reduces host fitness in the absence of the enemy (Figure 2A, h = 0), and hence does not satisfy the general definition of mutualism in such conditions. However, the situation changes in the presence of enemies because now also a costly symbiont may improve host fitness in relation to symbiont-free hosts (Figure 2A, h = 1). Accordingly, Fellous & Salvaudon [9] have framed the concept of “conditionally helpful parasites”, referring to parasites providing beneficial fitness effects for a host in some special conditions and being harmful in others. These cases are congruent with the concepts of symbiotic relativism [20] and mutualism-parasitism continuum [29], where the ecological outcome of symbiosis is seen to vary from parasitic to mutualistic among different environments.

## Materials and Methods

### Study system

The fungus *Claviceps purpurea* has over 400 host species within the family Poaceae, including rye and other economically important cereals and forage grasses. Ergot is common in temperate climates and it occurs also in subtropical and arctic regions [30], [31]. Ergot infection is restricted to a single ovary/seed, but one or several other seeds in the inflorescence may carry sclerotia due to separate primary infections or secondary infections by conidia present in “honeydew” produced by the fungus in the early stage of infection [14].

We used red fescue, *Festuca rubra,* as the host plant and domestic sheep as the herbivore in order to roughly estimate the costs and possible defensive effects of ergot infection in nature. *F. rubra* is a common grass species in the wild as well as in agricultural habitats in northern Scandinavia, and is frequently infected with ergot (Wäli et al., unpublished). The sheep pastures studied are semi-natural meadows dominated by *F. rubra* and utilized for rotational grazing. In rotational grazing animals are moved from pasture to pasture during the grazing period. Thus, the pasture vegetation is not fully consumed and grasses may flower and produce seed during and outside grazing periods.

### Effect of ergot on red fescue seed number

*F. rubra* individuals are mainly perennial and long-lived clonal colonies formed of several ramets flowering in different years. Therefore, we used the quantity of seeds produced per ramet (inflorescence), a fitness correlating measure, to estimate the cost of ergot infection. We collected mature inflorescences of *F. rubra* from seven semi-natural meadows and one riverside population in northern Finland in September 2008 and 2009 (Appendix S1). Depending on the size and infection intensity of the population, four to 20 ergot-infected and uninfected inflorescences were collected randomly from the population. The number of seeds, ergots and empty florets were recorded from each inflorescence, constituting the total amount of florets per inflorescence. The full florets were dissected to reveal possible small-sized sclerotia.

### Effect of grazing on ergot infection frequencies

The effect of grazing on ergot infection frequencies was estimated by sampling *F. rubra* inflorescences from sheep pastures in northern Scandinavia (Appendix S2). We collected 24–61 samples from each of 6 separate locations in September 2006 and 2008. Inflorescences were collected at random from moderately grazed sheep pastures and from corresponding ungrazed areas outside the pasture fence. Inside the pastures the overall amount of *F. rubra* inflorescences was diminished due to grazing and thus the density of *F. rubra* inflorescences differed between the grazed and ungrazed sites.

### Sheep reaction to ergot containing feed

To test whether large herbivores avoid, or in the short term learn to avoid eating ergot, we conducted food choice experiments with domestic sheep at MTT Agrifood Research Finland, Animal Production Research, Jokioinen. The single-day experiment was carried out indoors in a sheep shed in December 2009. The preference for ergot-containing and ergot-free forage was estimated in a pairwise test. Ergot sclerotia of rye (*Secale cerale*) were mixed with forage pellets in a 1∶4 volume ratio. Half litres of ergot-containing and ergot-free control pellets were offered on similar plastic trays placed side by side on the floor. A single sheep (male, n = 6) was allowed to approach the trays at a time, and was allowed to choose between the trays one to four times. Sheep were allowed to visually examine, smell and touch the feeds, but a metal fence placed above the pellets prevented actual eating. In the test, the actions of positive choice (sheep trying to eat forage) or rejecting the feed (after visual or other cue) were recorded. Activity was recorded as a positive choice (eating decision) when sheep kept their head in the tray, actively trying to eat for over 3 seconds, and the test was continued each time until the sheep selected one of the trays. Six rounds of the test were conducted, and the arrangement of trays was changed randomly between animals.

### Ethical statement

The sheep used in this research were experimental animals of Animal Production Research of MTT Agrifood Research Finland, Jokioinen, kept with institutional permits in accordance with The Finnish Act on Animal Experimentation. The national Animal Experiment Board of Finland was consulted, and no specific permits were required for the food choice test described in this study, as the sheep were not let to ingest ergot, and thus the test did not meet the criteria for an animal experiment described in The Finnish Act on Animal Experimentation (62/2006) as "carrying out such experiments, tests, research or investigations on animals. which may cause pain, suffering, distress or lasting harm comparable at least to the pain caused by the introduction of a needle". No protected or endangered species were used in the field studies, and private owned land was accessed with landowners' permission.

### Statistical analyses

The logistic regression (binomial distribution and logit link function) of events/trials data was employed to three separate tests to estimate 1) whether the proportional ergot sclerotia amount in inflorescences differed among eight wild grass populations (population as fixed factor) and 2) whether successful seeds from all florets differed among these grass populations and among grass inflorescences with and without ergot infection (population and ergot infection as fixed factors). We tested further 3) whether the number of ergot sclerotia as continuous variable affected the proportion of successful seeds from all florets in inflorescences. In this case, the scale parameter was estimated by the square root of deviance/dof.

To compare ergot infection frequency of *F. rubra* inflorescences in grazed and ungrazed areas and among sites, the data of ergot incidence in grass inflorescences was analysed as event/trial data with logistic regression (binomial distribution and logit link function), the event being the presence of one or more ergot sclerotia in a *F. rubra* inflorescence examined and the trial being a *F. rubra* inflorescence collected and examined. Grazing (two levels) and collection site (six levels) were used as fixed factors in the model.

The food choice data was analysed as event/trial data with logistic regression (binomial distribution and logit link function), the event being the positive food choice and the trial being the approach of the forage tray. We tested the effects of each three factors (ergot, sheep individual and test round) in the food tray choice of sheep in separate analyses due to the low number of replicates. Because positive food choice was recorded every time when the control tray was approached, the data of approaching the ergot containing tray was used to test the differences among sheep individuals. In case of test rounds, the scale parameter was estimated by the square root of deviance/dof.

The analyses were performed using SAS 9.3, with the GENMOD procedure.

## Results

### Effect of ergot on red fescue seed production

In wild grass populations the proportional ergot sclerotia content in inflorescences did not differ among eight wild grass populations in Finnish Lapland (χ^2^ = 6.44, *P*<0.17). The overall number of flowers and the seed production (number of seed) varied greatly: in ergot infected inflorescences the seed number varied between 0 and 69, and in uninfected between 0 and 73, and the total amount of flowers per inflorescence was between 17 and 152 in infected and between 22 and 139 in uninfected inflorescences. The proportional seed production varied significantly between populations (χ^2^ = 41.61, *P*<0.0001) (see Appendix S1 for means and confidence intervals in seed production and ergot content in inflorescences in each population examined). Presence of ergot infection had no significant effect on successful seed production (χ^2^ = 1.20, *P* = 0.27), but the increase in proportion of ergotic florets (as a continuous variable) decreased the proportion of successful seed (χ^2^ = 4.45, *P* = 0.035).

### Effect of grazing on ergot infection frequencies

Ergot infection incidence in red fescue inflorescences differed among grazed and ungrazed areas (χ^2^_1_ = 26.18, *P<*0.0001) and among collection sites (χ^2^_5_ = 51.24, *P<*0.0001). Frequency of ergot infected inflorescences was higher in pastures (43 %) than surrounding non-grazed areas (16 %). Ergot infection frequencies of grazed and ungrazed areas in different collection sites are presented in Appendix S2.

### Sheep reaction to ergot containing feed

**Figure 3:**
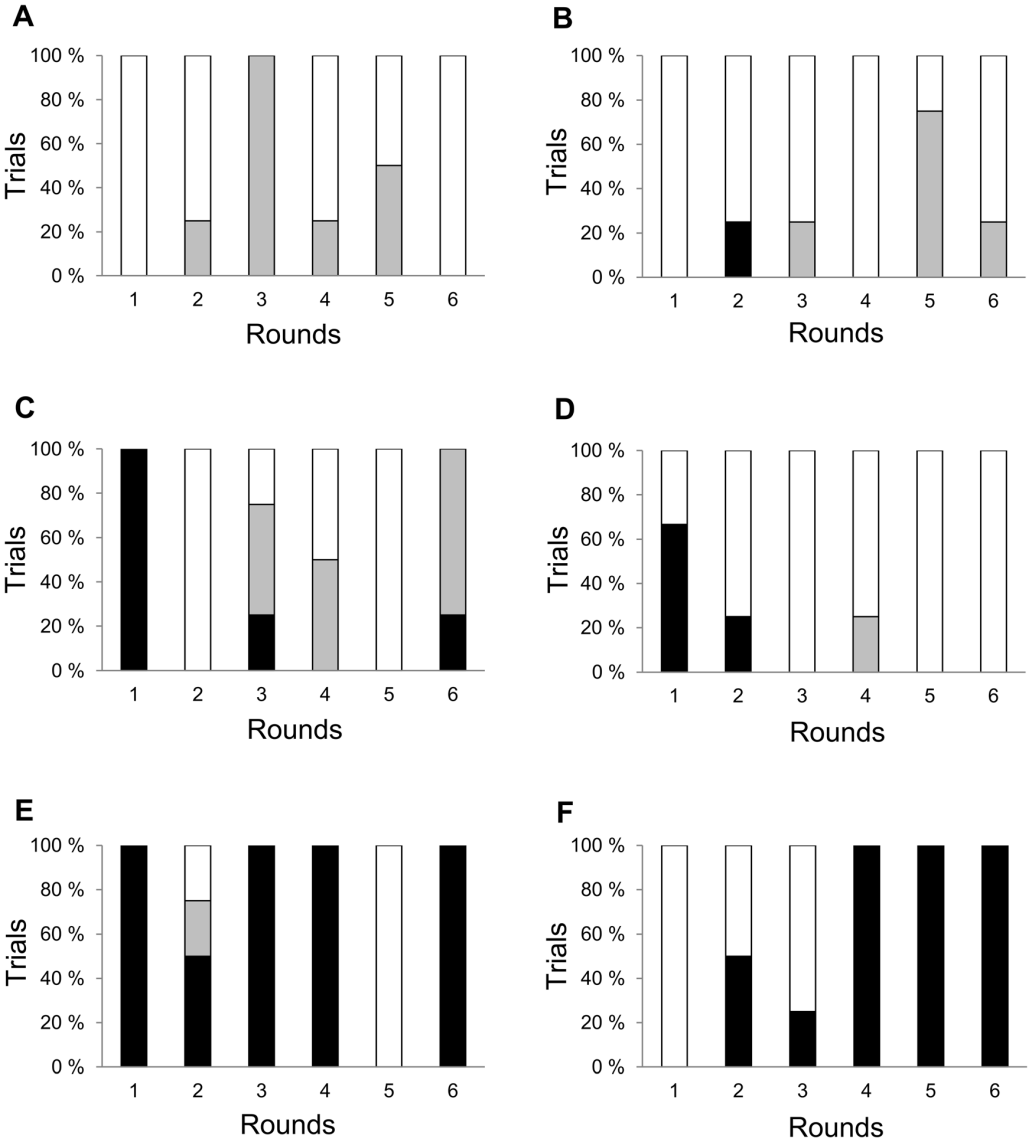
Individual sheep reactions to ergoty feed. Single rams' (A–F) actions relating to ergot-containing feed on different rounds of a food choice test. The column colours indicate the proportion of actions within trials: white, ergoty feed not approached; grey, ergoty feed rejected; black, ergoty feed chosen.

Sheep actions at the ergot-containing tray differed from actions at the control tray (χ^2^ = 47.53, *P<*0.0001). All the sheep made positive choice (eating decision) every time they approached the control tray, but some avoidance was detected with the ergot-containing tray. Actions did not differ significantly between rounds (χ^2^ = 3.32, *P = *0.65), but sheep individuals differed in their reactions (χ^2^ =  48.64, *P = *0.0001). Four of the six rams clearly selected against ergot-containing feed, when two nearly always made the positive choice from the tray they first approached (Figure 3).

**Figure 4:**
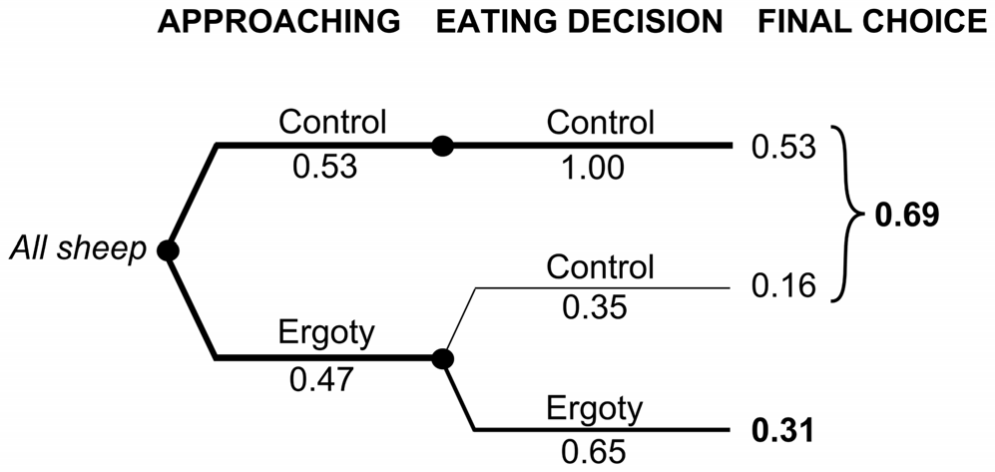
Overall reaction of sheep to ergoty feed. A decision tree representing the average probabilities of rams' (n = 6) approaches and decisions between ergoty and control forage in a food choice test. The final choice signifies the alternative eventually chosen for eating.

When taking averages over rounds of individual sheep and over six individuals, the animals had an almost equal probability of approaching control or ergoty trays (Figure 4). The most pronounced difference was found concerning how the rams responded to food quality. When approaching the control tray, the sheep always made a positive choice and never shifted away to the ergoty tray. In contrast, when they first approached the ergoty tray, they chose that tray only in 65 % of cases, and shifted to the control tray in 35 % of cases. Because of the shift from the ergoty to the control tray, control food was selected more often (69 %) as compared to 31 % of the ergoty food.

Concerning the variability in the average responses between individual sheep, individual A slightly more frequently (62 % cases) approached the control tray and always rejected the ergoty food (Figure 3). On the other hand, individuals E and F approached more frequently (78 % and 65 %, respectively) the ergoty tray and nearly never changed to another tray (Figure 3).

## Discussion

Our model predicts that, in spite of possibly losing some seeds due to infection, a host plant may benefit from the ergot-provided protection against grazers, and by these means save more seeds than it loses in the presence of grazers. Our empirical observations confirm that this hypothesis is plausible. First, the seed loss of a ramet caused by the infection is rather small or even non-existent if only a few sclerotia per inflorescence are produced. Secondly, the ergot frequencies were higher in sheep pastures than surrounding ungrazed areas. This difference may well be the consequence of selective foraging, where sheep have favoured uninfected flower heads and avoided infected ones. This is in accordance with the results of the food choice experiment, where sheep often changed from a sclerotia-containing feed to a sclerotia-free alternative and never *vice versa*.

### Direct costs and host control over infection

According to our field data, ergot may not self-evidently cause marked direct costs to its host. By contrast, clearly negative effects of ergot on grain and seed production (number and weight of grain/seed) have been reported with agricultural crops and Kentucky bluegrass (*Poa pratensis*) grown for seed [32], [33]. However, the results from cultivated grasses and cereals may not correspond to the situation in nature, due to the selective breeding, genotypic uniformity of host populations and artificial, rather constant growth conditions, like high density and high nutrient concentrations [2], [3], [34]. With wild salt marsh *Spartina* species, negative, but similarly to our results, also neutral and even positive effects of ergot infection on seed set have been reported, depending on the infection level, host species and/or different habitats [35], [36].

In addition to seed number, the total costs of ergot infection may include additional effects, like reduced weight, quality and viability of uninfected seeds in the inflorescence [32], [37]. We did not test for the effects on viability of seeds or seedling establishment in this research, but in studies with wild *Spartina foliosa* and cultivated Kentucky bluegrass ergot infection caused no effect on seed germination, although Kentucky bluegrass seed storability was somewhat impaired [32], [36].

The seemingly minor costs of ergot infection may be due to several factors: e.g. host resistance and tolerance to ergot, offering the host ways to control and compensate for the infection. Resistance can be seen as factors limiting the chance and extent of infection (e.g., [38], [39]). *C. purpurea* has an exceptionally wide range of hosts including the entire subfamily Pooideae [30]. Even though being of great interest, finding completely resistant cultivars or varieties of grass and cereal ergot host species has not been very successful. No specific resistance genes have been detected in cereal crops [40], but genetic variability in ergot resistance has been found e.g. in Kentucky bluegrass and rye [37], [41]. This indicates that there has not been strong selection for resistance to ergot in the pooid family, further implying that the net costs of ergot infection for the host are not high in general.

Instead, host grasses may have adapted the ability to restrict ergot infection to a tolerable level. This is in accordance with our results, as the infected inflorescences most commonly bore only one or two sclerotia. One mechanism of restriction could be the escape of infection by asynchronous flower development within grass inflorescences, as it is known to affect, for example, seed predation [42]. The differences of ergot resistance in cereals indicate the potential to limit the size and the resources consumed by an ergot sclerotium. The detected low level of ergot infection within grass inflorescences may also alternatively or partly result from various environmental factors such as weather and available sources of infection.

Further, grasses seem to have a high tolerance to ergot, for example, the ability to mitigate or offset the adverse fitness effects of ergot infection (e.g., [38], [39]). According to Jaroz & Davelos [43], a floral infection may not directly reduce plant reproductive effort because plants can often produce more flowers than mature seeds. Such was the case with our data, as the seed production of wild red fescue (when compared to the flower amount) was overall very low and not all florets produced seed. Thus, ergot may not actually always reduce seed number by taking a place of a seed, and the host may have the potential to allocate resources to uninfected flowers instead of infected ones.

Salvaudon et al. [13] have speculated about reproductive compensation, and even overcompensation, of a plant for a predictable attack by parasites, similarly to the compensation for herbivory. With ergot this could be via delaying the development of florets and/or using resources limitedly in populations with high pressure of pathogen attack. “In case” of ergot infection grasses might then be able to allocate spared resources to reproduction within, or also between inflorescences, since many perennial grass species produce several flowering shoots at different times during a growing season. Accordingly, Raybould et al. [35] have proposed that ergot could encourage seed set by causing changes in the host's resource allocation by increasing the investment to flower heads and seed production at the expense of other plant functions. A similar mechanism is suggested to cause some positive effects of closely ergot-related *Epichloë* endophytes on seed production during some life stages of its host grasses [44], [45]. The physiological mechanism for this change in resource allocation may be due to fungal compounds acting like plant hormones, as some fungal endophytes may produce auxin-like plant-growth regulators [46].

### Herbivory and indirect benefits of infection

We found clear differences in overall ergot infection frequencies between moderately grazed pastures and surrounding ungrazed areas. The higher infection incidence in pastures indicates that grazing somehow affects the proportion of ergot-infected inflorescences. One probable explanation could be selective grazing preferring the uninfected grass inflorescences with ergot-infected ones left uneaten. Documenting actual plant selection in the field by grazing animals is needed for a more accurate confirmation of the exact level of host tiller and genet protection by ergot.

Our food choice experiment supported the hypothesis of selective grazing, as the overall choice against ergot-containing forage was significant. The sheep individuals however differed in their actions, with four out of six rams clearly avoiding the ergot-containing forage, and two always trying to eat from the container they first approached. The variance in behaviour among individuals, which is common in choice experiments on learning behaviour (e.g., [47]), may be due to the individual characters and abilities, either in adjusting to short-term experimental settings or in the ability to detect and/or avoid ergot. With the latter, there may also have been differences in ergot-related history between the rams, e.g. previously encountering ergot in the pasture. The animals were also not allowed to actually eat the forages in this test, so possible aversion learning must have taken place earlier.

Mammalian herbivores are most likely able to distinguish the presence of ergot. Mammals can detect and avoid endophyte-infected plants, even by their alkaloid profile [48]. This is possibly due to the bitter taste of alkaloids [19], and ergot sclerotia can contain high doses of similar alkaloids [16], [19], [49]. Ergot is also claimed to have a distinctive smell detectable even by man (rev. in [23]). Moreover, the fully developed dark-coloured sclerotia of *C. purpurea* are usually larger than seeds and often curved, which makes them clearly visible when protruding from florets. Lev-Yadun and Halpern [25] suggested that the colour of ergot could even provide a visual aposematic signal for mammalian herbivores. The authors also speculated about the possibility of development of food aversion towards ergot-infected grasses. Ingestion of ergot sclerotia in low levels is not lethal, but can cause symptoms which make development of food aversion possible [25]. Bakau & Bryden [50] have detected avian discrimination of ergoty food in a longer-term food choice study in which birds had the possibility to also ingest ergot.

With our data, it is impossible to propose the actual mechanism for ergot avoidance. However, from a plant's perspective, the reason and mechanism of ergot-induced avoidance is irrelevant. If the presence of ergot negatively affects the seed predation probability of an inflorescence, the plant gains a protective effect. Accordingly, ergot may well provide defence for other tillers of the host plant, and even for neighbouring plants.

In our model, we have assumed that ergot infection causes direct loss of seeds, and hence beta (i.e. the relative decline in viable seeds per plant if infected by ergot) can achieve only positive values. The argument can be expanded so that beta may also result in negative values indicating that ergot-infected plants produce more seeds than non-infected plants. In such a case ergot would be beneficial to the plant without any protective effects. In fact, herbivores could theoretically even be slightly attracted by ergot infected plants and still ergot would be beneficial to the host as far as the loss of seeds due to the greater risk of grazing is smaller than the overall improved seed production of ergot infected plants. In this study we found only a very low cost associated with ergot infection in terms of seed number per inflorescence, so this scenario might be possible in some ergot-grass species combinations.

### Ergot – a conditionally helpful pathogen

In this study, we only refer to fitness effects on the host on the ecological time scale. The alkaloids produced by ergot, which are the cause for the host-acquired chemical defence, have probably evolved to protect the fungus itself, and have a protective effect on the host as a by-product. Such a case can be defined as “by-product mutualism” [7]. Regarding the evolutionary history of the relationship, our study is not sufficient to claim that the interacting species have evolved special adaptations to receive “mutualistic benefits” from each other (see [6], [8]). Such a coevolved mutualistic symbiosis is most easily developed when transmission of the symbiont is vertical (e.g. *Epichloë* grass endophytes), whereas according to some species interaction models in horizontally transmitted symbionts, like ergot, higher virulence would evolve more easily (e.g., [51]). On the other hand, mutualism is commonly detected among free-living species, e.g. among ectomycorrhizal fungi and plants as well as pollinating insects and plants, and thus, evidently, vertical transmission is not a necessary condition for mutualism to evolve [52]. Actually each plant is a part of a complex web of interacting mutualists, both vertically transmitted symbionts and free-living organisms, coevolving together [53].

Fellous & Salvaudon [9] have discussed the evolution of parasitic interaction into a mutualistic relationship in relation to conditionally helpful parasites. They suggest, that if the beneficial fitness effect is strong, even the rarely occurring mutualistic situation may select against resistance to the parasite infection. According to Fellous & Salvaudon [9], the interaction may evolve towards mutualistic symbiosis especially if the parasite provides a completely new trait or function for the host. Ergot-grass interaction demonstrates this scenario, since grasses usually do not have chemical defence mechanism against herbivores, but cope with herbivores by tolerating grazing [19] and with weak phytolith (silica bodies) defence [54]. Toxic ergot provides the host with a new trait, the acquired chemical defence, compared to uninfected grasses. Grasses seem not have evolved strong resistance against ergot, which indicates the lack of straightforward selection against ergot infection. In many grassland ecosystems grasses experience recurrent grazing, which is the situation where grasses would benefit from any additional protection against herbivory.

We conclude that ergot infection involves direct costs to the host grass, but the costs may not necessarily be notable in wild grass populations. Ergot may provide the host with indirect, ecological benefits via acquired chemical defence in conditions were mammalian herbivores are present. Thus, we suggest that the ergot-grass system is an example of conditional pathogen-host interaction in which the outcome may fall on different parts of a continuum from parasitism to mutualism depending on the specific situation. The interaction may be defined as conditional defensive mutualism.

The protective effect provided by ergot is concentrated on reproduction and reproductive tissue, inflorescence and the seed set, where the fitness effects are most pronounced. This is the case especially in conditions where large herbivores consume most of the inflorescences. In fact, the defensive effect of ergot has acted against human exploitation, as heavily affected seed and grain sets have been, and still are, disposed of. Our argument could partly explain why grasses are commonly susceptible and rather tolerant to ergot and no effective resistance mechanisms have evolved over time. This might provide new insight into plant breeding programs of pooid cereals, where ergot resistance is one of the goals.

## Supporting Information

Appendix S1 **Seed production of ergot infected and uninfected red fescue ramets.** Location, coordinates, habitat, collection dates and mean proportion of seeds in ergot-infected and ergot-free inflorescences in each grass population.
(DOCX)

Appendix S2 Ergot incidence in red fescue inflorescences in sheep pastures and corresponding ungrazed areas. Location, coordinates, habitat, collection year, ergot infection frequency and sample size.
(DOCX)
