# Supplementary material for: Is the Pathogenic Ergot Fungus a Conditional Defensive Mutualist for Its Host Grass?
Source: PLoS One. 2013 Jul 10;8(7):e69249. doi: 10.1371/journal.pone.0069249 (PMC3707848; doi:10.1371/journal.pone.0069249)
Supplement: Appendix S1 — Seed production of ergot infected and uninfected red fescue ramets. Location, coordinates, habitat, collection dates and mean proportion of seeds in ergot-infected and ergot-free inflorescences in each grass population. (DOCX) [file pone.0069249.s001.docx]

Appendix S1. Seed production of ergot infected and uninfected red fescue, *Festuca rubra*, inflorescences in eight grass populations in northern Finland.

| *Site* | *Location* | *Coordinates* *(WGS84) lat/lon* | *Habitat* | *Collection date* | *Mean proportion of florets with seed in ergot infected inflorescences* | *Mean proportion of florets with seed in ergot free inflorescences* |
| --- | --- | --- | --- | --- | --- | --- |
| Niemelä | Utsjoki, Finland | 69° 54.937'/ 27° 5.207' | Fresh meadow | 6.9.2009 | 28% | 41% |
| Utsjoki | Utsjoki, Finland | 69° 54.297'/ 27° 1.519' | Old meadow | 6.9.2009 | 33% | 44% |
| Kutuniemi | Utsjoki, Finland | 69° 45.767'/ 27° 0.597' | Old meadow | 6.9.2009 | 32% | 28% |
| Tsieskula | Utsjoki, Finland | 69° 44.238'/ 27° 1.279' | Old meadow | 6.9.2009 | 41% | 36% |
| Puksala | Utsjoki, Finland | 69° 43.916'/ 27° 1.211' | Old meadow | 6.9.2009 | 46% | 41% |
| Kotkapahta | Utsjoki, Finland | 69° 44.436'/ 26° 59.086' | Rocky river bank | 2008 | 7% | 4% |
| Valkkojärvi | Inari, Finland | 69° 4.303'/ 27° 20.992' | Forest meadow | 3.9.2009 | 18% | 18% |
| Solojärvi | Inari, Finland | 68° 50.149'/ 26° 45.822' | Old meadow | 2.9.2009 | 23% | 27% |
