# Supplementary material for: Is the Pathogenic Ergot Fungus a Conditional Defensive Mutualist for Its Host Grass?
Source: PLoS One. 2013 Jul 10;8(7):e69249. doi: 10.1371/journal.pone.0069249 (PMC3707848; doi:10.1371/journal.pone.0069249)
Supplement: Appendix S2 — Ergot incidence in red fescue inflorescences in sheep pastures and corresponding ungrazed areas. Location, coordinates, habitat, collection year, ergot infection frequency and sample size. (DOCX) [file pone.0069249.s002.docx]

Appendix S2. Ergot incidence in *F. rubra* inflorescences in six sheep pastures and corresponding ungrazed areas in northern Scandinavia.

| *Site* | *Location* | *Coordinates* *(WGS84) lat/lon* | *Habitat* | *Collection year* | *Ergot infection frequency, grazed area* | *Number of inflorescences collected, grazed area* | *Ergot infection frequency, ungrazed area* | *Number of inflorescences collected, ungrazed area* |
| --- | --- | --- | --- | --- | --- | --- | --- | --- |
| Kevonsuu | Utsjoki, Finland | 69° 45.530'/ 26° 59.349' | Semi-natural meadow | 2006 | 68% | 37 | 20% | 15 |
| Jeesiö | Sodankylä, Finland | 67° 30.483'/ 26° 2.629' | Meadow | 2006 | 18% | 34 | 0% | 17 |
| Rajala | Sodankylä, Finland | 67° 35.095'/ 26° 26.678' | Meadow | 2008 | 20% | 30 | 5% | 21 |
| Bonjágas | Tana, Norway | 70°43.739'/ 28°24.359' | Meadow | 2006 | 58% | 12 | 64% | 11 |
| Álletnjárga | Tana, Norway | 70°09.901'/ 28°14.488' | Meadow | 2008 | 69% | 29 | 22% | 32 |
| Ándejohka | Tana, Norway | 70°09.784'/ 28°14.626' | Meadow | 2006 | 28% | 18 | 0% | 16 |
